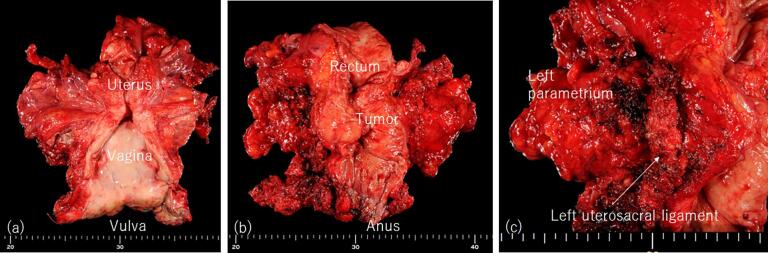# Transperineal endoscopic approach with GelPOINT V-path in laparoscopic pelvic exenteration for postirradiated recurrent cervical cancer

**DOI:** 10.1016/j.gore.2023.101291

**Published:** 2023-10-07

**Authors:** Hiroyuki Kanao, Motoko Kanno, Atsushi Fusegi, Yoichi Aoki, Makiko Omi, Terumi Tanigawa, Sanshiro Okamoto, Hidetaka Nomura

**Affiliations:** Department of Gynecologic Oncology, Cancer Institute Hospital, 3-8-31 Ariake, Koutouku, Tokyo 135-8550, Japan

**Keywords:** Dead pelvic space, Laparoscopic pelvic exenteration, Prior radiotherapy, Recurrent cervical cancer, Transperineal endoscopic approach

## Abstract

Pelvic exenteration (PE) is a highly invasive procedure associated with high morbidity and mortality rates. Laparoscopy is a promising option to reduce this invasiveness, and laparoscopic PE significantly reduces blood loss and shortens hospital stays. In the case of a large tumor with invasion to the surrounding organs, laparoscopic dissection around the pelvic floor is sometimes problematic owing to restrictions on handling instruments. To overcome these limitations, we performed a transperineal endoscopic approach using the GelPOINT V-path in addition to laparoscopic PE. This approach enabled dissection around the pelvic floor without the abovementioned obstacles under magnified visualization. As a result, we could dissect the pelvic floor precisely with a reduction of the dead pelvic space, which might contribute to reduced rates of postoperative complications while ensuring oncologic outcomes.

## Highlights

• Laparoscopic dissection around the pelvic floor is sometimes problematic owing to restrictions on handling instruments.
• The transperineal endoscopic approach enabled the preservation of the left iliococcygeal muscle.
• The transperineal endoscopic approach facilitated minimal dissection of the ischiorectal fossa.

## 1 Introduction

Pelvic exenteration (PE) is the only curative treatment in patients with post-radiation recurrent cervical cancer. Since 1948, when PE was first reported by Dr. Alexander Brunschwig, acceptable technical feasibilities and acceptable oncological outcomes have been reported (Brunschwig, 1949). However, the morbidity rate associated with this procedure is approximately 70 %; therefore, improvements in surgical techniques are required (Peiretti et al., 2012).

Recently, several reports have described that compared with an open procedure, laparoscopic PE seems to be advantageous regarding blood loss and hospital stay, with similar oncologic outcomes (Casey et al., 2022). Thus, laparoscopic PE for recurrent gynecological cancer is an effective alternative to open procedures.

However, laparoscopic approaches are sometimes problematic in bulky tumors on the pelvic floor because of restrictions placed on handling instruments. Patients who undergo laparoscopic PE have large tumors often due to invasion of the surrounding organs, and precise handling of the forceps at the pelvic floor is difficult. Consequently, during laparoscopic PE, unnecessary dead space tends to develop around the pelvic floor (Lei et al., 2018). Since a pelvic dead space can lead to serious postoperative complications such as pelvic abscess, bowel obstruction, and fistula (Sutton et al., 2022), a novel approach is required to reduce the dead space in laparoscopic PE.

The GelPOINT V-path is a novel surgical instrument developed as a platform for Transvaginal Natural Orifice Transluminal Endoscopic Surgery (vNOTES), which enables endoscopic procedures in a narrow vagina (Goldenberg et al., 2020). In this case, we performed transperineal endoscopic surgery with the GelPOINT V-path in addition to laparoscopic PE in a patient with postoperative recurrent cervical cancer. We successfully minimized the pelvic dead space, potentially leading to a reduction in postoperative complications.

## 2 Case report

**Supplementary Fig. 1:**
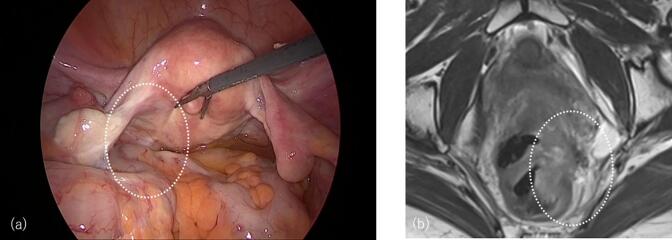


A 55-year-old patient with stage IIIB cervical mucinous carcinoma (gastric type) underwent concurrent chemoradiotherapy; however, one year later, the tumor recurred as a solitary lesion in the pelvis. The recurrent tumor occupied the left uterosacral ligament and perforated the rectum (Fig. S1). Pelvic exenteration with laterally extended endopelvic resection (LEER) of the left side was planned to complete the recurrent tumor resection.

**Supplementary Fig. 2:**
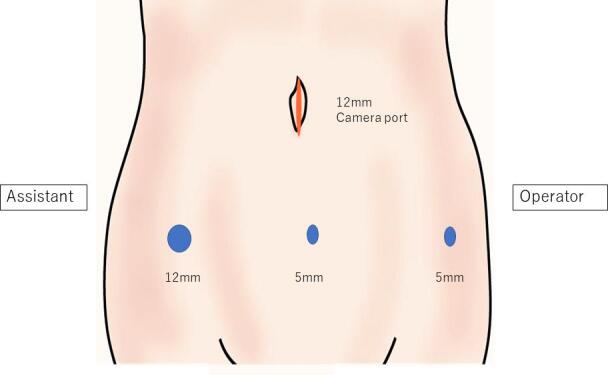


A laparoscopic procedure with four trocar placements was initiated (Fig. S2). A uterine manipulator was not used to prevent cancer cell spillage.

**Supplementary Fig. 3:**
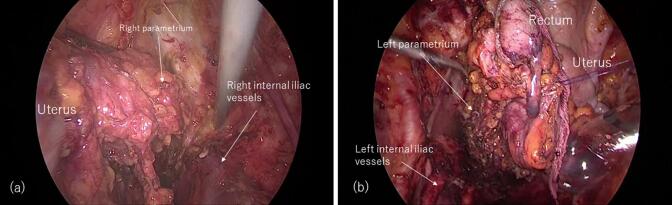


The uterus, rectum, bladder, and ureters were in one mass and fixed to the left pelvic sidewall. The pararectal and paravesical spaces were developed, and the cardinal ligaments were observed. After dividing the right ureter, the right cardinal ligament was divided at the internal iliac vessels (Fig. S3a). The left ureter was divided, the internal iliac artery and vein, S2, piriformis muscle, and sacrospinous ligament were resected, and LEER was successfully performed on the left side (Fig. S3b). This procedure detached the tumor from the pelvic sidewall with sufficient surgical margins.

Next, we dissected down to the pelvic floor.

**Fig. 1:**
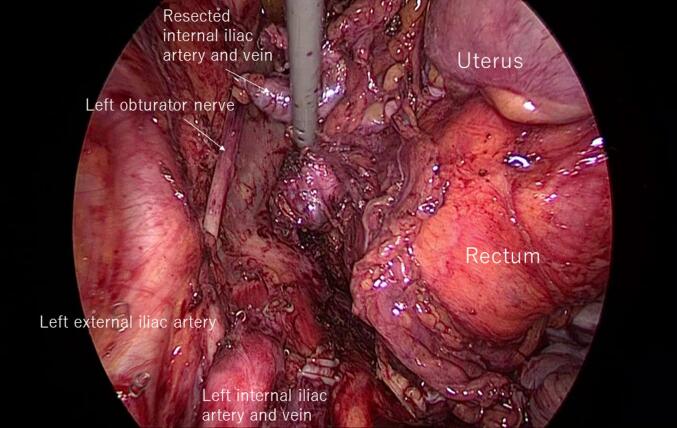
Surgical view of the left pelvic floor (LEER side). The surgical field on the left pelvic floor was poor owing to the overhanging tumor, and the forceps interfered with the tumor.

Because the resected pelvic organs, including the recurrent tumor, were large and overhanging on the left side, the movement of the laparoscopic forceps on the left pelvic floor was restricted (Fig. 1). Consequently, the ischiorectal fossa on the left side inevitably developed widely via the laparoscopic approach, and we changed to a transperineal maneuver.

**Fig. 2:**
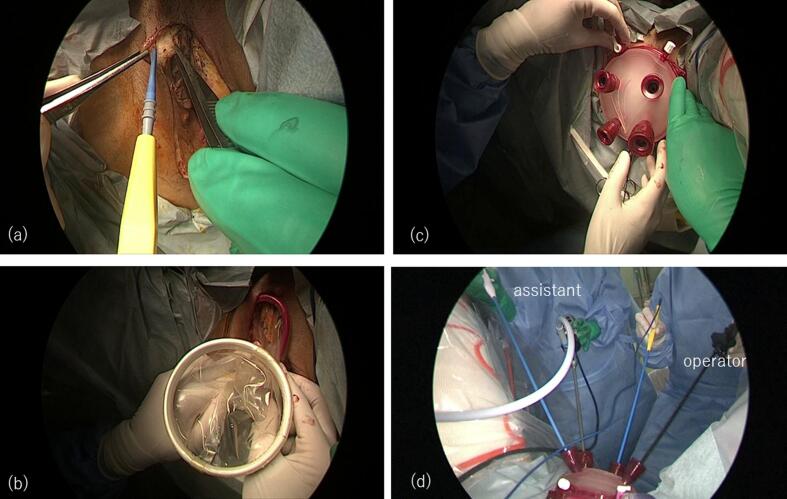
Placement of GelPOINT V-path at the vulva. (**a**) A skin incision was made at the outer labia majora, and the subcutaneous tissue was dissected. (**b**) A 9.5 cm Alexis retractor was attached. (**c**) Four trocars were placed at the Alexis retractor. (**d**) Operation scene.

**Supplementary Fig. 4:**
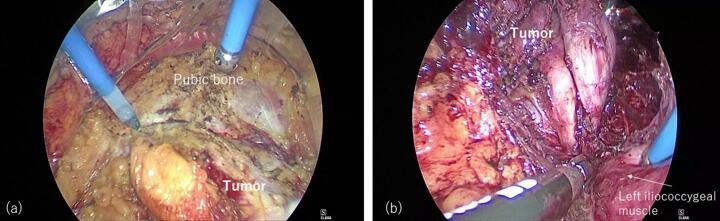


After the anus was closed with silk sutures, a skin incision was made at the outer labia majora, and the subcutaneous tissue was dissected (Fig. 2a). A purse-string suture was placed with a 1–0 PDS at the perineal incision, and a 9.5 cm Alexis retractor was attached (Fig. 2b). Four trocars were placed on the Alexis retractor (Fig. 2c, d). The ischiorectal fossa developed via the perineal endoscopic approach (Fig. S4a, b), and reached the abdominal cavity in line with the laparoscopic dissection, and total pelvic exenteration could be conducted.

**Fig. 3:**
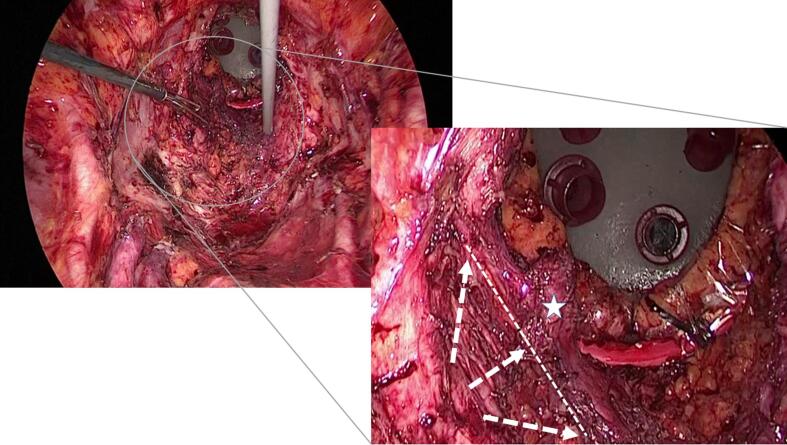
The pelvic view after retrieval of the specimen. The transperineal endoscopic approach enabled the preservation of the left iliococcygeal muscle and minimal dissection of the ischiorectal fossa. Arrow: Laparoscopic dissection line. Dotted line: Estimated resection line of the pelvic floor by laparoscopic approach only. Star: Preserved iliococcygeal muscle of the left side.

The operative view after retrieval of the specimen is shown in Fig. 3. On the left side of the pelvic floor, where the tumor was located, laparoscopic dissection was performed on the outer side of the left iliococcygeal muscle. However, the transperineal endoscopic approach enabled the preservation of the left iliococcygeal muscle and minimal dissection of the ischiorectal fossa.

A colostomy and an ileal conduit were constructed with a small incision of 7 cm. The minimal dead pelvic space in the ischiorectal fossa could be filled completely with a right-sided gluteal fold flap.

Surgery was performed without any intraoperative complications. The operation time was 8 h 48 min, and the blood loss volume was 340 mL without blood transfusion. Pathology confirmed that the tumor was completely resected, with circumferential resection margins of >2 mm (Fig. S5a,b,c).

There were no postoperative complications, including pelvic abscess or bowel obstruction, and the patient had no fever after the fifth postoperative day. The patient could have been discharged about a week after the surgery. However, the discharge occurred on the twenty-first postoperative day, after removing the ureteral stent, because the patient did not want to be discharged with the urinary stent in place.

The patient received no adjuvant treatment, and there was no sign of recurrence six months after surgery.

Three patients underwent laparoscopic PE with transperineal endoscopic surgery using the GelPOINT V-path. All procedures were safely performed without intraoperative complications, and complete resection was achieved. There were no cases of postoperative pelvic abscess or bowel obstruction.

## 3 Discussion

When a large dead space is created in the pelvis during PE, postoperative complications, such as pelvic abscess, bowel obstruction, and fistula formation due to the fall of the small intestine into the dead space, are problematic (Sutton et al., 2022). One solution is to reduce dead space by filling it with biomaterials (Devulapalli et al., 2016). Many studies have reported the usefulness of omental and myocutaneous flaps (Miyamoto et al., 2016). Although an omental flap is easy and safe, the omentum is often resected during the initial surgery for gynecological cancers. Even if it remains, it is usually insufficient to fill the dead space.

Consequently, myocutaneous flaps are commonly used to fill the dead pelvic space during PE. An abdominal flap such as the vertical rectus abdominis myocutaneous (VRAM) flap has been reported to be the most effective; however, this approach may not be feasible in patients requiring multiple ostomies. Perineal and thigh-based flaps such as the gluteal fold flap, gluteus maximus musculocutaneous flap, and gracilis myocutaneous flap are the treatment choices; however, these flaps may be limited by their arc of rotation and less reliable skin paddle, which can be problematic in larger perineal defects (Copeland-Halperin et al., 2020).

In this case,

The volume of the omentum was small and insufficient to fill the dead pelvic space.
Double ostomies (sigmoid colostomy and ileal conduit) were required, and an abdominal-based flap (such as a VRAM flap) was not feasible.
The left internal iliac artery and vein were divided to secure sufficient surgical margins; therefore, a perineal and thigh-based flap on the left side was not feasible.

Therefore, we decided to fill the dead space with a gluteal fold flap on the right side only. As a result, it was necessary to reduce the dead space because the volume of the right myocutaneous flap was small.

Laparoscopic PE is an alternative treatment option to decrease blood loss and shorten hospital stays (Casey et al., 2022). However, when laparoscopic PE is performed in patients with a bulky tumor, the pelvic floor around the tumor may be dissected more widely than necessary because of the restriction of forceps movement. A transperineal approach is conventionally used to overcome these restrictions. This “caudal-to-cranial” maneuver can perform deep pelvic dissection without the abovementioned obstacles; however, the surgical field for conventional perineal maneuvers is narrow and deep, and some blind dissection must be performed, which leads to unnecessary bleeding or incorrect dissection. The transperineal endoscopic approach using the GelPOINT V-path enables deep pelvic dissection under magnified visualization, which can reduce unnecessary blood loss. Controlling intraoperative bleeding enables surgeons to dissect the appropriate layer, facilitating complete resection. In such cases, complete resection with negative surgical margins can be performed without blood transfusions. A recent meta-analysis showed that the transperineal endoscopic approach for rectal surgery offers advantages such as reduced blood loss, shorter hospital stays, and lower postoperative complication rates (Lei et al., 2018).

Robotic surgery allows forceps to bend flexibly, and robotic forceps can move without restriction, even when dissecting the pelvic floor around a bulky tumor. In this case, robotic surgery may have reduced the dead space without requiring a perineal approach. However, robotic surgery remains very expensive and only available in some institutions. The GelPOINT V-path is a familiar surgical instrument developed for gynecological surgery.

This is the first report on using GelPOINT V-path for a transperineal approach in a patient with post-radiation recurrent cervical cancer. This novel approach is technically feasible.

Finally, no previous randomized controlled trials (RCTs) have compared open and laparoscopic exenteration. However, considering that the LACC trial suggested an increased risk of recurrence with minimally invasive radical hysterectomy, RCTs are warranted to demonstrate the oncologic safety of laparoscopy for recurrent cervical cancer.

## 4 Conclusion

We encountered a case in which transperineal endoscopic surgery using the GelPOINT V-path minimized pelvic dead space during laparoscopic PE. Although further studies are needed in many cases, this novel approach may reduce the incidence of postoperative complications such as pelvic abscess, bowel obstruction, and fistula formation during laparoscopic PE.

**Ethics statement:** IRB approval was obtained (IRB number: 2023-GB-034).

**Informed consent:** Written informed consent was obtained from the patient.

## CRediT authorship contribution statement

**Hiroyuki Kanao:** Writing – review & editing, Writing – original draft, Visualization, Software, Resources, Methodology, Formal analysis, Data curation, Conceptualization. **Motoko Kanno:** Writing – review & editing, Investigation. **Atsushi Fusegi:** Writing – review & editing, Validation, Supervision. **Yoichi Aoki:** Writing – review & editing, Project administration, Investigation. **Makiko Omi:** Writing – review & editing. **Terumi Tanigawa:** Writing – review & editing. **Sanshiro Okamoto:** Writing – review & editing. **Hidetaka Nomura:** Writing – review & editing.

## Declaration of Competing Interest

The authors declare that they have no known competing financial interests or personal relationships that could have appeared to influence the work reported in this paper.

## Figures and Tables

**Supplementary Fig. 5:**